# Elucidating the molecular bases of epigenetic inheritance in non-model invertebrates: the case of the root-knot nematode *Meloidogyne incognita*

**DOI:** 10.3389/fphys.2014.00211

**Published:** 2014-06-06

**Authors:** Laetitia Perfus-Barbeoch, Philippe Castagnone-Sereno, Michael Reichelt, Sara Fneich, David Roquis, Loris Pratx, Céline Cosseau, Christoph Grunau, Pierre Abad

**Affiliations:** ^1^INRA, Institut Sophia Agrobiotech, UMR 1355 ISASophia-Antipolis, France; ^2^CNRS, Institut Sophia Agrobiotech, UMR 7254 ISASophia-Antipolis, France; ^3^Institut Sophia Agrobiotech, Université de Nice Sophia-Antipolis, UMR ISASophia-Antipolis, France; ^4^Abteilung Biochemie, MPI für Chemische ÖkologieJena, Germany; ^5^Ecologie et Evolution des Interactions, Université de Perpignan Via DomitiaPerpignan, France; ^6^Ecologie et Evolution des Interactions, CNRS, UMR5244Perpignan, France

**Keywords:** epigenetics, *Meloidogyne incognita*, chromatin, DNA-methylation, histone modification

## Abstract

Root-knot nematodes of the genus *Meloidogyne* are biotrophic plant parasites that exhibit different life cycles and reproduction modes, ranging from classical amphimixis to obligatory mitotic parthenogenesis (apomixis), depending on the species. *Meloidogyne incognita*, an apomictic species, exhibits a worldwide distribution and a wide host range affecting more than 3000 plant species. Furthermore, evidences suggest that apomixis does not prevent *M. incognita* from adapting to its environment in contrast to what is expected from mitotic parthenogenesis that should theoretically produce clonal progenies. This raises questions about mechanisms of genome plasticity leading to genetic variation and adaptive evolution in apomictic animals. We reasoned that epigenetic mechanisms might in part be responsible for the generation of phenotypic variants that provide potential for rapid adaptation. We established therefore a pipeline to investigate the principal carriers of epigenetic information, DNA methylation and post-translational histone modifications. Even if *M. incognita* possesses the epigenetic machinery i.e., chromatin modifying enzymes, 5-methyl-cytosine and 5-hydroxy-methyl-cytosine content is absent or very weak. In contrast, we demonstrated that the canonical histone modifications are present and chromatin shows typical nucleosome structure. This work is the first characterization of carriers of epigenetic information in *M. incognita* and constitutes a preamble to further investigate if *M. incognita* development and its adaptation to plant hosts are under epigenetic control. Our pipeline should allow performing similar types of studies in any non-model organism.

## Introduction

Epigenetics is the study of heritable changes in gene expression and function that cannot be explained by changes in DNA sequence. These molecular mechanisms can be influenced by the environment and the individual history as well as being potentially transferable from a generation to the next, with a reversible character (Youngson and Whitelaw, 2008). Organisms are forced to adapt to the heterogeneousness of the environment and the phenotypic plasticity constitutes a major adaptation to compulsory constraints. Since the system of reproduction of an organism conditions its evolutionary dynamics as well as the demography and the spatial genetic structuring of its populations, it is of particular relevance to study epigenetic variation in asexually reproducing organisms. Indeed, in spite of a genetic homogeneity in asexually reproducing organisms, an important phenotypic variability can be observed between individuals of the same clonal lineage found in different environments. The influence of the epigenetic mechanisms could bring an explanation to this paradox (reviewed in Verhoeven and Preite, 2014). Similarly, epigenetic modifications may provide an accessory source of fast-acting, reversible, and readily available phenotypic variations that can shape host–pathogen interactions (reviewed in Gómez-Díaz et al., 2012). Despite growing evidences on the role of epigenetic phenomena on a wide range of biological phenomena, many questions remain for the involvement of epigenetics in plasticity and adaptation.

Root-knot nematodes (RKN) of the genus Meloidogyne are obligatory plant parasites that constitute major agricultural pests worldwide. They infect almost all cultivated plants and establish an intimate interaction with their hosts inducing the re-differentiation of root cells into hypertrophied and multinucleate feeding cells (Caillaud et al., 2008). So far, the most efficient control measure against RKN was the use of chemical substances called nematicides. However, because of their toxic side effects on the environment, nematicides have recently been banned from use. Novel approaches to control them are sorely needed. Among RKN species, some are strict or facultative amphimitic while others reproduce exclusively by mitotic parthenogenesis (apomixis) (Castagnone-Sereno, 2006). Our model, *Meloidogyne incognita*, reproduces in an asexual way by parthenogenesis without meiosis. Genetically identical individuals develop from *M. incognita* females and form virtually clonal populations. Although these clones share the same genetic heritage, modifications of their phenotype can be observed when they are exposed to unfavorable environments (Castagnone-Sereno et al., 1994). These phenotypes can be completely different such as juveniles molting into either female or male, but also show for instance a switch of virulence leading to infection of resistant hosts. A first step toward a better understanding of plant-nematode interaction was the sequencing of the genome of *M. incognita*. Analysis of this reference genome highlighted characteristics, which could be at the origin of the parasitic success of this species (Abad et al., 2008; Danchin et al., 2010).

Epigenetics changes refer to a set of molecular processes that can affect gene expression such as regulatory processes mediated by remodeling of chromatin structure through methylation of cytosine residues in the DNA and chemical modifications of histone proteins, in particular their acetylation or methylation (Jablonka and Lamb, 2002). On one hand, DNA methylation refers to a chemical modification of genomic DNA by the addition of a methyl (-CH3) group to specific nucleotide bases. The most common form of DNA methylation is cytosine methylation, occurring predominantly in CpGs in animal genomes (Suzuki and Bird, 2008). However, because of lack of *de novo* methylation machinery, DNA methylation process is unlikely to occur in nematodes, including *C. elegans* but with the exception of *Trichinella spiralis* (Simpson et al., 1986; Wenzel et al., 2011; Gao et al., 2012, 2014). On the other hand, post-translational modifications of histone amino acid side chains (PTMs) are known to mediate epigenetic regulation of gene expression and development in animals (Li et al., 2007; Luger et al., 2012; Turner, 2012). For instance, PTMs, such as acetylation of histone H3 at lysine 9, are typically found in euchromatin and often associated with active transcription, whereas other modifications, such as methylation of lysine 9 or lysine 27 of histone H3, are found in heterochromatin and related to gene repression (Jenuwein and Allis, 2001). One major role for epigenetic variation in evolution would be to promote phenotypic variability, and allow populations to widely explore new environmental conditions. This could lead to a rapid adaptation to environmental changes or colonization of new environments. The interactions between parasites and their host are models of choice to study these mechanisms because the selective pressures are strong and the evolution is fast. *M. incognita* constitutes an ideal model to study these mechanisms, especially as clones can be obtained following the naturally occurring asexual multiplication in host plants. Whereas variation at the genetic level has been studied in *M. incognita*, less is known about the extent and function of epigenetic variation. Previous analyses on multiple *M. incognita* populations have shown that genomic polymorphism could not be directly correlated to observed phenotypes (Semblat et al., 2000).

Many non-model organisms of ecological and economical importance such as *M. incognita* suffer from a lack of even rudimentary knowledge about their epigenetic information carriers. To address this problem, we describe here a straightforward 2 × 3-step procedure to perform a fundamental analysis of elements of the chromatin marking system. We first performed a whole genome bioinformatics search for chromatin-modifying enzymes such as those involved in DNA methylation, we evaluate the degree of DNA methylation by calculating the CpG observed/expected ratio in *M. incognita* transcriptome and by measuring DNA methylation levels of 5-methylcytosine (5-mC) and 5-hydroxy methylcytosine (5-hmC) in genomic DNA by LC-MS/MS. We then used Western blots to verify the presence of histone and histone modifications and tested critical parameters of a standardized N-ChIP procedure that need to be adapted for each model.

## Analysis of DNA methylation

DNA methylation is a common feature of many but not all genomes. We recommend starting with this feature.

### Step 1: *in-silico* searches of chromatin-remodeling enzymes

To understand whether *M. incognita* possesses the capacity to methylate DNA, we first conducted reciprocal BLASTp searches combined to OrthoMCL analysis to identify proteins that might be related to known chromatin-remodeling enzyme orthologs (Table 1). Significant hits were defined as those satisfying the following criteria: *E*-value <10^−5^ and the aligned segments covering at least 30% of the sequence length of the hit. OrthoMCL has been applied to the proteome data set from *M. incognita* and three publicly available nematode genomes (*C. elegans*, *T. spiralis*, and *Pristionchus pacificus*). Animal DNA methyltransferases can be subdivided into three subfamilies, DNMT-1, DNMT-2 and DNMT-3, based on sequence similarity (Kumar et al., 1994; Hendrich and Tweedie, 2003). DNMT-1 has been regarded as a “maintenance” methyltransferase, whereas DNMT-3 functions as “*de novo*” methyltransferase and some evidence suggest that DNMT-2 enzymes are active cytosine-5′-methyltransferases (Kunert et al., 2003). Species exhibiting functional DNA methylation generally encode a complete set of DNMTs in their genomes whereas species lacking DNA methylation, such as *C. elegans*, have lost DNA methylation enzymes from their genomes (Gutierrez and Sommer, 2004; Suzuki and Bird, 2008). BLASTp search of the *M. incognita* whole proteome (Supplementary Material) was performed with DNMT-1, DNMT-2, DNMT-3 protein sequences from nematodes (Table 1): the *Trichinella spiralis* DNMT-1 (accession number EFV58204.1); *P. pacificus* Ppa- DNMT-2 (accession number AY766101.1) and *T. spiralis* DNMT-22 (accession number EFV60295.1); *T. spiralis* DNMT-3 (accession number EFV54759.1). As for the 11 other nematodes in which DNA methylation has been studied, with exception of *T. spiralis* (Gao et al., 2012), *M. incognita* did not possess *de novo* methylation machinery, DNMT-3 nor DNMT-2. However, our data revealed the existence of a potential DNMT-1 orthologous protein annotated in *M. incognita*, as well as methyl-CpG binding domain (MBDs) proteins, which encodes another essential component of the methylation system (Table 1). We found that Minc01117 could be ortholog to the maintenance methyltransferase DNMT-1 from other species. Sites of DNA methylation are occupied by various proteins, including MBD proteins, which recruit the enzymatic machinery to establish silent chromatin. Nematodes such as *C. elegans* (Cel-MBD-2, accession number C27A12.10), *C. briggsae* (Cbr-MBD-2, accession number CBP11474) and *P. pacificus* (Ppa-MBD-2, accession numberAY766102.1) all contain an mbd-2-like gene (Gutierrez and Sommer, 2004). We found that two predicted *M. incognita* proteins, Minc14299 and Minc14778, could be orthologs to the MBD-2 like protein.

**Table 1:** ***M. incognita* putative orthologs for methyltransferases (DNMTs) and methyl-CpG binding domain (MBDs) proteins**.

**Protein**	**Species**	**Accession number**	***M. incognita* putative orthologs**
**DNA METHYLTRANSFERASES (DNMTs)**			
DNMT-1	*Trichinella spiralis*	EFV58204.1	Minc01117
DNMT-2	*Trichinella spiralis*	EFV60295.1	*M. incognita* absent from OrthoMCL group
	*Pristionchus pacificus*	AY766101.1	
DNMT-3	*Trichinella spiralis*	EFV54759.1	*M. incognita* absent from OrthoMCL group
**METHYL-CpG BINDING DOMAIN 2 (MBDs)**			
Cel-MBD-2	*Caenorhabditis elegans*	C27A12.10	Minc14299 Minc14778
Cbr-MBD-2	*Caenorhabditis briggsae*	CBP11474	
Ppa-MBD-2	*Pristionchus pacificus*	AY766102.1	

### Step 2: CpG observed/expected ratio (CpGo/e) analysis

**Figure 1:**
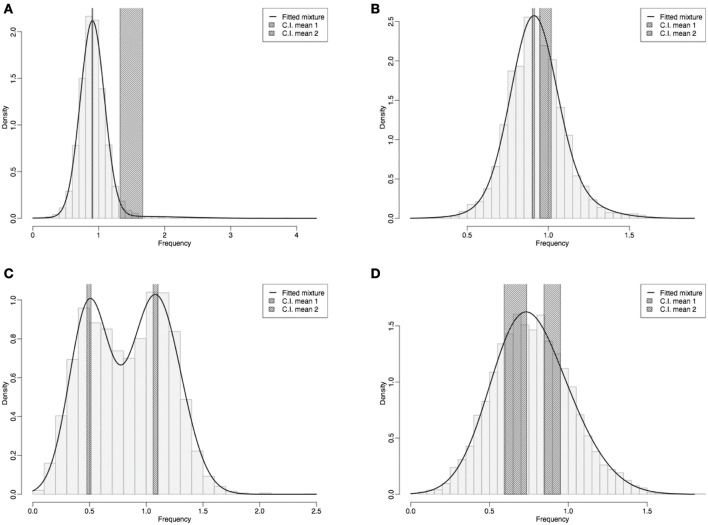
**Frequency distribution of CpG observed/expected ratio (CpGo/e) in four different species**. **(A)** *C. elegans* and **(B)** *D. melanogaster*, and on **(C)** *A. melifera* and **(D)** *M. incognita*. CpGo/e ratio was measured as a proxy to estimate the CpG methylation in transcripts from EST data. X axis: CpGo/e ratio, Y-axis density (frequency distribution) of ESTs. The figure displays a histogram of Bg GUA CpGo/e ratios with a fitted mixture distribution. The gray shaded bars represent 95% confidence intervals for the two mean values.

To evaluate the degree of DNA methylation, we calculated the empirical distribution of CpGo/e ratios for *M. incognita* transcriptome. A total of 63,838 expressed sequenced tags (EST) of *M. incognita* that were downloaded from NCBI-dbEST public database (Supplementary Material), filtered by size (≥500 bp) leading to 41,649 sequences of which 5000 were randomly chosen for CpG observed/expected ratio (CpGo/e) analysis. Most of the data came from *M. incognita* strain Morelos maintained at INRA Sophia Antipolis and recently published (Abad et al., 2008; Jaouannet et al., 2012). To validate the robustness of CpGo/e ratio method, the same experiment was conducted on *C. elegans* (initially 8722 ESTs), the model nematode lacking DNA methylation process, *D. melanogaster* (38,110 ESTs) and on *A. mellifera* (10,157 ESTs). *D. melanogaster* has been previously described for its lack of DNA methylation at late stages of development (Urieli-Shoval et al., 1982; Lyko et al., 2000) while DNA methylation occurs in *A. mellifera* (Elango et al., 2009). The CpGo/e ratio was used as a proxy to estimate the intragene DNA methylation content as previously described in *Biomphalaria glabrata* (Fneich et al., 2013). In *C. elegans*, the CpGo/e ratio profile exhibited an unskewed Gaussian distribution with an estimated mean value close to 1 (0.93 ± 0.26) as expected for species lacking such process (Figure 1A). A similar result was obtained with *D. melanogaster* late stage ESTs (Estimated mean value 0.92 ± 0.17; Figure 1B). In contrast, the CpGo/e ratio in *A. mellifera* transcripts can be approximated by a mixture of two Gaussian distributions (Figure 1C). This pattern has also been described in the mollusc *B. glabrata* where transcripts are divided in low and high methylated genes (Fneich et al., 2013). *M. incognita* CpGo/e ratio profile did not perfectly fit *C. elegans* profile (Figure 1D). In *M. incognita*, the CpGo/e ratio profile also exhibited a skewed near-Gaussian distribution but with a slight shift on the left compared to *C. elegans* profile. The estimated mean value for *M. incognita* transcriptome was inferior to 1 (0.77 ± 0.25). However, careful analysis of the curve fitting suggests the presence of two populations of sequences: one with a mean value around 0.9 and another with a mean value around 0.7. These results could indicate the presence of two populations of genes in *M. incognita*: one with no methylation and one with very low methylation.

### Step 3: global DNA methylation analysis by LC-MS/MS

Genomic DNA used for liquid chromatography-mass spectrometry analysis was prepared from *M. incognita* eggs with the CTAB method (Winnepenninckx et al., 1993) and stored at −80°C until use. The levels of 5-methylcytosine (5-mC) and 5-hydroxy methylcytosine (5-hmC) were determined by liquid chromatography-mass spectrometry LC-MS/MS analysis as their deoxyribonucleosides as described in (Fneich et al., 2013). Neither 5-methyl-cytosine nor 5-hydrox-methyl-cytosine could be detected.

Taken together, our results show that DNA methylation is absent or very low in *M. incognita.*

## Analysis of nucleosomes, histones, and histone modifications

In the absence of DNA methylation, PTMs could represent one of the primary epigenetic transcriptional control mechanisms. Such a process has been shown to play a key role in the regulation of gene transcription of parasites, e.g., the apicomplexan family of protozoa and the metazoan *Schistosoma* (Mourão et al., 2012; Perrin et al., 2013). The importance of these modifications in regulating diverse developmental programs in parasites motivated us to study their importance in *M. incognita* in the framework of this study. Experiments were performed on *M. incognita* strain “Morelos” which have been previously used for the whole genome sequencing of this nematode (Abad et al., 2008). Because *M. incognita* is an obligatory plant parasite, it has to be maintained on plants to complete its life cycle. We used tomatoes, *Solanum esculentum* cultivar Saint Pierre, grown at 20°C in a greenhouse as *M. incognita* host for this study. Moreover, samples that we collected are considered to be clonal because of the mitotic parthenogenetic mode of reproduction of *M. incognita* and since the lab population was originated from the progeny of a single female. To grow and produce nematodes, one-month-old tomato plants are inoculated with *M. incognita* second-stage juveniles (J2s). As previously described by Rosso et al., 1999, eggs were collected from 7 week-old infected tomato roots after grinding, sterilizing (0.5% NaOCl) and filtering steps. Extracted eggs were kept either to hatch J2s or to be aliquoted and stored at −80°C for further experiments (e.g., genomic DNA extraction and chromatin immuno-precipitation). To produce J2s, extracted eggs were kept on 10 μm sieve in autoclaved tap water, aerated with an air pump, at room temperature. After 5 days, J2s hatched and were collected by centrifugation (13000 g, 1 min). J2 samples were stored at −80°C for further experiments (e.g., Western blot and chromatin immuno-precipitation).

### Step 1: detection of canonical histones and histone modifications by western blots

The nucleosome is subject to a dizzying array of posttranslational modifications, which work alone or in combination to constitute a histone code that regulates chromatin structure and function (Jenuwein and Allis, 2001). The four core histones, H2A, H2B, H3, and H4, which are responsible for folding DNA into nucleosomes, have been described as very highly conserved throughout evolution (Mariño-Ramírez et al., 2006). This high conservation allowed us to test commercial antibodies that react with mammals but also with a wide range of species. In some species, chromatin is condensed around the centromere and characterized by several methyl groups on lysine amino acid (K) at 9 and 20 position of histone H3 and H4 respectively. These marks are noted H3K9Me2/3 and H4K20Me3 and can be recognized by specific antibodies. Before immuno-precipitation of *M. incognita* DNA, we tested 12 commercial antibodies, directed against histone H3 and several H3 and H4 modifications, on *M. incognita* J2s with western blots.

Western blots were performed as previously described by Azzi et al. (2009). Briefly, about one thousand *M. incognita* J2s were re-suspended in denaturation buffer, treated by sonication and boiled 5 min at 99°C. Proteins were separated by SDS-PAGE and transferred to nitrocellulose membranes (Amersham RPN203D) by the semi-dry method (SEMI-PHOR Bio-Rad). The membrane was blocked overnight at 4°C in blocking buffer and incubated with one of the 12 following commercial antibodies (1μg/μl): Anti-histone H3 (H3, abcam ab1791 and Active Motif 39164); Anti-Histone H3 dimethylated at lysine 4 (H3K4Me2, Abcam ab32356) or at lysine 36 (H3K36Me2, Abcam ab9049); Anti-Histone H3 trimethylated at lysine 4 (H3K4Me3, Millipore 04-745 and Abcam ab8580), at lysine 9 (H3K9Me3, Abcam ab8898 and Upstate) or at lysine 27 (H3K27Me3, Diagenode pAB-069-050); Anti-Histone H3 acetylated at lysine 9 (H3K9Ac, Upstate); Anti-Histone H4 trimethylated at lysine 20 (H4K20Me3, abcam ab9053) and Anti-hyperacetylated Histone H4 (H4PentaAc, Upstate). Bands were revealed by Enhanced Chemical Luminescence (ECL Pierce) and direct exposure to X-ray film (Amersham).

**Figure 2:**
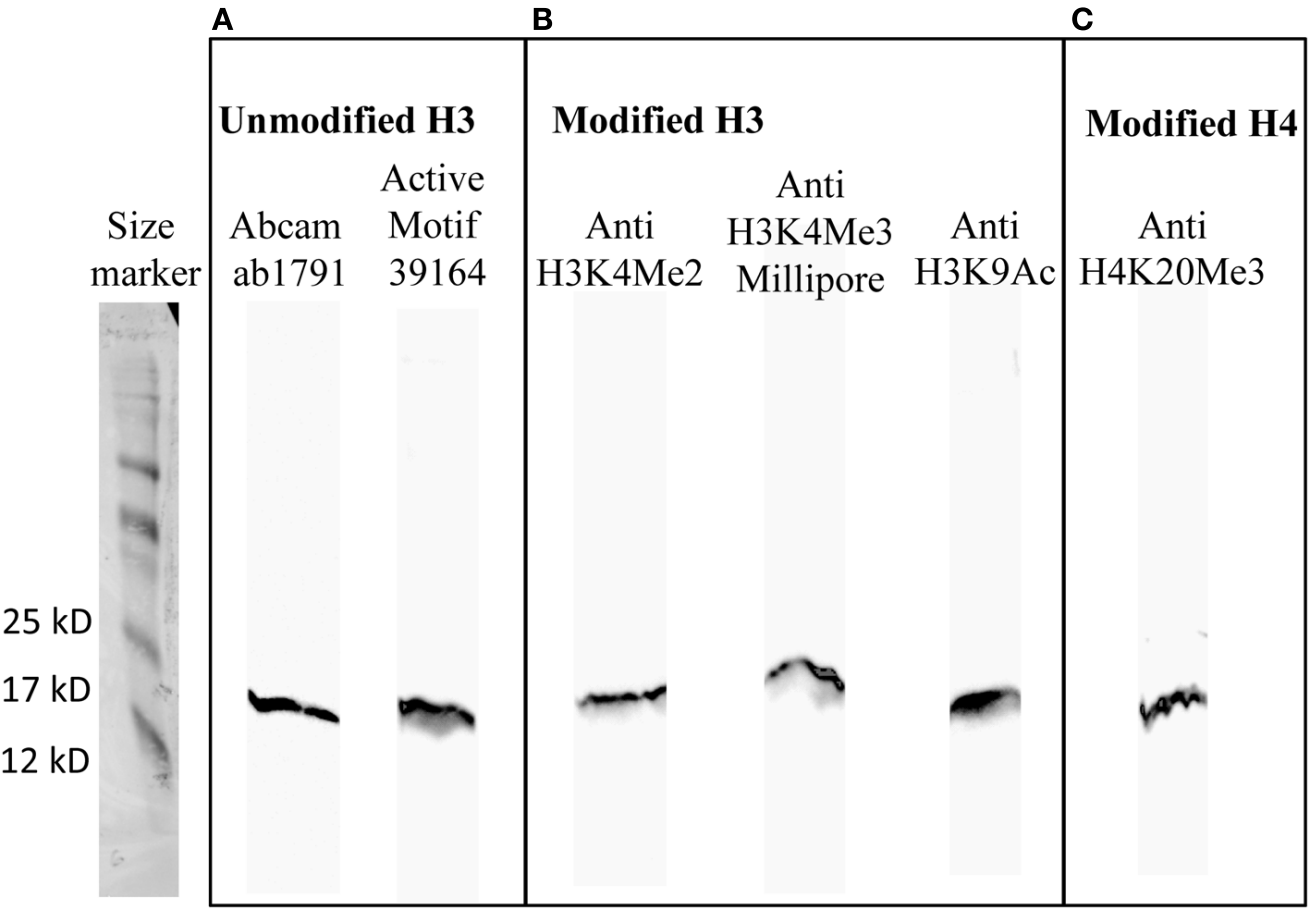
**Western blot detection of (A) unmodified histone H3, (B) modified histone H3: dimethylated H3 at lysine 4 (H3K4Me2) and acetylated H3 at lysine 9 (H3K9Ac) and (C) modified histone H4: trimethylated H4 at lysine 20 (H4K20Me3) in *M. incognita* J2s**.

The antibodies should give a unique staining on the membrane (one unique band) to be selected for DNA immuno-precipitation. Three antibodies, Anti-H3K4Me3 (Abcam), Anti-H3K9Me3 (Abcam and Upstate), did not give any signal (Supplementary Figure 2A). One antibody, Anti-H4PentaAc gave a non-specific signal with many high molecular weight bands (Supplementary Figure 2B). Two antibodies, Anti-H3K27Me3 and Anti-H3K36Me2 gave two distinct bands (Supplementary Figure 2C). Six antibodies, Anti-histone H3 (Abcam and Active), Anti-H3K4Me2, Anti-H3K4Me3 (Millipore), Anti-H3K9Ac and Anti-H4K20Me3 gave a unique signal at 17 kDa (Figure 2) as expected for histones (Bártová et al., 2005).

We conclude that canonical histones and histone modifications are present and that six commercially available antibodies could be used for immuno-precipitation of *M. incognita* chromatin.

### Step 2: controlled fragmentation into nucleosomes

**Figure 3:**
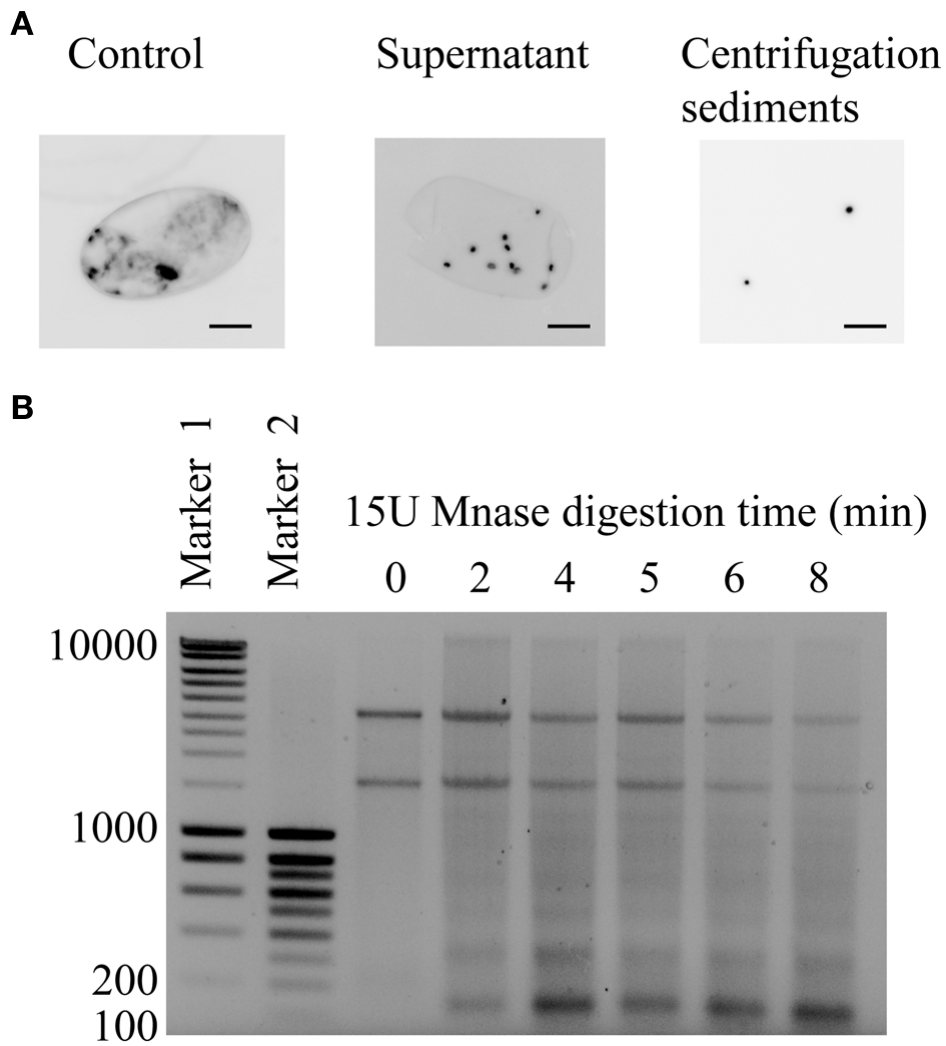
**(A)** Pictures showing nucleus (in black; Hoechst staining) of *M. incognita* eggs observed under microscope (40X, Nikon T1-SM) before (Control) and after grinding (Supernatant and Centrifugation sediments). The experiment started with intact eggs (Control) that were grinded for 5 min in Dounce homogenizer to release nuclei. After centrifugation, only nuclei were observed in centrifugation sediments whereas nuclei and fragments remained in supernatant. Scale bar, 10 μm. **(B)** Electrophoresis gel of *M. incognita* eggs DNA after 0, 2, 4, 5, 6, and 7 min MNase (15 U) treatment showing a very characteristic pattern, similar to a ladder, for nucleosome. Marker 1 and 2 exhibit regularly spaced bands ranging from 200 to 10,000 bp and 100 to 1000 bp, respectively.

Because little is known about chromatin organization in *M. incognita*, we started by testing partial Micrococcal Nuclease (MNase) digestion of chromatin to reveal its nucleosome structure, which is the fundamental unit of chromatin. Because DNA portions of nucleosome core particles are less accessible for MNase than linking sections, DNA gets digested into fragments of lengths equal to multiplicity of distance between mono-nucleosomes. First, to access nuclei, we tested different grinding times, from 3 to 10 min, for cell lysis in Dounce homogenizer. As a measure for grinding efficiency, we colored nuclei using Hoechst, a fluorescent stain that binds strongly DNA. Also, because *M. incognita* life-stages are really different in shape and composition, we tested these grinding conditions on both J2s and eggs. Best results were obtained with 10 min total in Dounce homogenizer, distributed as 5 min grinding following by 5 min at rest (Figure 3A). Then, we tested different MNase concentrations (from 15 to 30 U; Figure 3B and Supplementary Figure 3) and incubation times (0, 2, 4, 5, 6, and 7 min). Typical mono-nucleosome structure was observed with fragments about 150 bp and multiplicity of this size (Figure 3B). Finally, 5 min incubation with 1 μl MNase (15 U) was chosen as the standard conditions to perform further steps of immuno-precipitation of the chromatin in *M. incognita.*

### Step 3: chromatin immuno-precipitation (ChIP)

To immunoprecipitate *M. incognita* DNA, we used the ChIP procedure adapted for non-model organisms by Cosseau et al. (2009). Each step of the procedure (Supplementary Material) had to be customized for *M. incognit*a. Basically, we tested different grinding times for cell lysis and different Micrococcal nuclease (MNase, Affymetrix, USB 70196Y) concentrations and incubation times (see Results). Nuclei were stained with Hoechst 33342 fluorescent stain (1/2000 dilution; Invitrogen H1399) and observed under microscope (40X, Nikon T1-SM) before and after grinding.

Because the antibody should be in excess over the histone of interest for ChIP, we tested protein/chromatin precipitation efficiency in *M. incognita* with increasing antibody volume ranging from 0 to 16 μl (1 μg/μl), for each of 3 tested antibodies (among the 6 antibodies that could be use): Anti-H3 (Abcam), Anti-H3K4Me3 (Millipore), and Anti-H4K20Me3. For each antibody concentration, DNA from the bound and unbound fractions was extracted with phenol/chloroform so we could quantify the sequences associated with each antibody by qPCR. In other species, histone marks such as H3K4Me3 were enriched around the transcription start sites (TSSs) of genes and were associated with active transcription (Du et al., 2013). For this reason, primers were designed to match genomic region close to TSSs of *M. incognita* housekeeping genes, Actin and GAPDH (Minc06773 and Minc12412, respectively; Table 2). Quantitative Real-time PCR (qPCR) analyses were performed using the LightCycler 2.0 system (Roche Applied Science) and LightCycler Faststart DNA Master SYBR Green I kit (Roche Applied Science). qPCR amplification was done in a final volume of 10 μl composed of 2.5 μl of immuno-precipitated chromatin, 1.5 μl H_2_O, 0.5 μl of each primer 10 μM and 5 μl of master mix. The following Light-Cycler run protocol was used: denaturation, 95°C, 10 min; amplification and quantification (repeated 40 times), 95°C for 10 s, 62°C for 5 s, 72°C for 16 s; melting curve, 60–95°C with a heating rate of 0.11°C/s and continuous fluorescence measurement, and a cooling step to 40°C. For each reaction, the cycle threshold (Ct) was determined using the “2nd derivative” method of the LightCycler 480 Software release 1.5. Primers were designed for this experiment with the Primer3 web-interface (Table 2). Quality and specificity controls were performed for each qPCR product (Supplementary Figure 1): amplification of a unique 150 bp band was verified by electrophoresis separation through a 2% agarose gel; amplicons were sequenced to verify that they matched expected sequence (GATC-biotech; Table 2); Primer efficiency was calculated based on *Ct* value for increasing genomic DNA concentration (from 0 to 1 ng/ul); Melting curve and negative first derivative of the melting-curve were recorded for each qPCR product to validate that only one product was amplified (one peak was observed).

**Table 2:** **Amplicon sequences and primer sequences designed for qPCR**.

**Name**	**Sequence**	**Length (bp)**	**Product size (bp)**
*M. incognita* GAPDH amplicon	CGTGCAGCGGTTGAGAAGGATACTGTCCAAGTTGTTGCTGTCAATGACCCGTTCATTGATCTTGACTATATGGTTTGGGGGAGACTTTCTATTAATAACTCCAATAACTTTTAGGTCTATATGTTTAACTATGATTCCACCCACGGACGC		
Mi_ChIP_GAPDH_F	CGTGCAGCGGTTGAGAAGGA	20	150
Mi_ChIP_GAPDH_R	GCGTCCGTGGGTGGAATCAT	20	
*M. incognita* Actin amplicon	AAGATGGATGAAGAGGTAGCCGCCCTTGTTGTGGATAATGGCAGTGGAATGTGCAAGGTTAGTTAAAAATGCCCTTTTTTCGATTTAAAGTTCTGTTGTTTTTTAAAGGCTGGATTTGCGGGTGATGATGCTCCTCGTGCCGTTTTTCCA		
Mi_ChIP_Actin_F	AAGATGGATGAAGAGGTAGCCGCCC	25	150
Mi_ChIP_Actin_R	TGGAAAAACGGCACGAGGAGCA	22	

The amount of target DNA recovered in the immune-precipitated fraction was quantified by calculating the percent input recovery (% IR) normalized with the percent input recovery obtained with the housekeeping gene as previously described by Cosseau et al. (2009).

**Figure 4:**
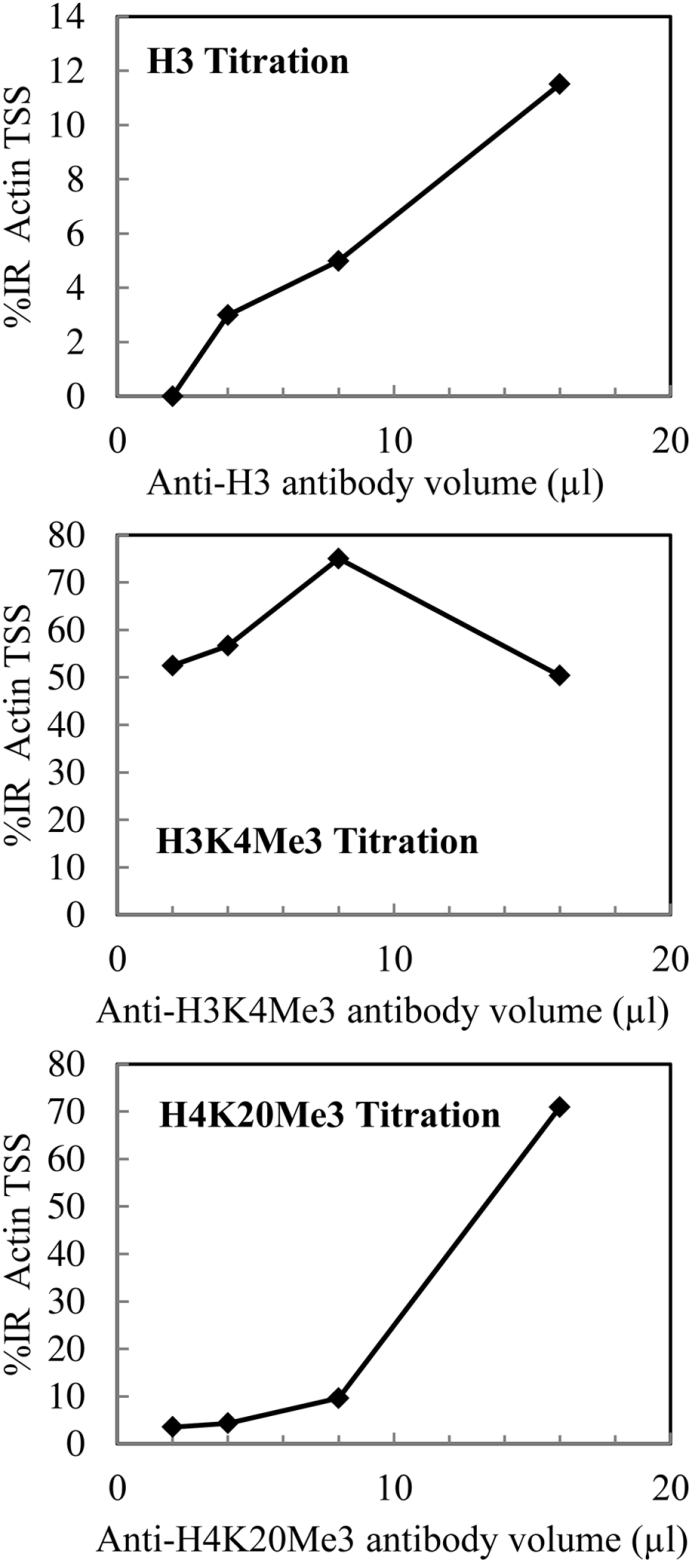
**qPCR on *M. incognita* chromatin that had been immuno-precipitated with various volumes (0–16 μl) of Anti-H3, Anti-H3K4Me3, and Anti-H4K20Me3 antibodies (1 μg/μl)**. The amount of target DNA recovered in the immune-precipitated fraction was quantified by calculating the percent input recovery (% IR) normalized with the percent input recovery obtained with the housekeeping gene as previously described by Cosseau et al. (2009).

Also, because of previous MNase digestion, the amplicon size has to be no longer than 150 pb to match *M. incognita* mono-nucleosome length. With *M. incognita* DNA, antibodies Anti-H3 and Anti-H4K20Me3 never reached saturation up to 16 μl (Figure 4). This could mean that these antibodies are not specific to histone H3 and histone H4 trimethylated at lysine 20 but recognize other proteins/marks. So, the more antibody was added, the more proteins interacted which generated a background signal detected thanks to qPCR. On the contrary, the Anti-H3K4Me3 antibody successfully immuno-precipitated *M. incognita* chromatin and reached saturation at 8 μl (Figure 4). Titration saturation for higher antibody concentrations indicates that the antibody is specific to the histone mark (Cosseau et al., 2009). Therefore, this antibody could specifically immuno-precipitate *M. incognita* and could be use for further analysis.

To summarize, we optimized the ChIP protocol for *M. incognita*, based on the previously published method for non-model organisms (Cosseau et al., 2009). First, we have optimized lysis and extraction of nuclei for *M. incognita* to 10 min grinding in digestion buffer. Then we found that the optimum time for MNase digestion (15U), to obtain fragmented chromatin into mono-nucleosome to penta-nucleosomes, a size range that is optimal for ChIP, was 5min. Finally, qPCR experiments indicated that N-Chip procedure should be performed with an excess of 8 μl antibody Anti- H3K4Me3. Now that chromatin immunoprecipitation has been optimized in *M. incognita* it opens new perspectives to further investigate the genome-wide distribution of PTMs by high-throughput sequencing (N-ChIP-Seq).

## Discussion

In the past few years, genome projects have demonstrated that DNA methylation is far more widespread than one would expect from the lack of DNA methylation in non-mammalian model organisms, such as the model nematode *C. elegans*. Moreover, functional DNA methylation has been recently reported in the parasitic nematode *T. spiralis* (Gao et al., 2012, 2014). Altogether, this makes us wonder what DNA methylation picture in *M. incognita* could be.

The *de novo* DNA methylation process can produce new methylated sites without pre-existing pattern by affecting cytosine nucleotide (Goll and Bestor, 2005). Depending on CG density, some of these de novo sites can then be maintained by DNMT maintenance methyltransferases. CpG under representation is generally seen as sign of DNA methylation (Bird, 1980). CpG o/e suggests that DNA methylation was present as a subset of genes in M. *incognita* ancestral species, that these sequences were obtained by horizontal gene transfer from a species with DNA methylation or that the EST database was contaminated. In the first case, *M. incognita* might have lost DNA methylation enzymes, which may have been present in the ancestral nematode, from its genome, as what is thought for *C. elegans* (Gutierrez and Sommer, 2004; Suzuki and Bird, 2008). Nevertheless, the observed CpG bias could also be due to other mechanisms (Jabbari and Bernardi, 2004). By LC-MS DNA methylation could not be detected in *M. incognita* that goes in line with the absence of gene coding for de novo DNA methylation activity.

The nucleosome is one of the most highly conserved structures known in eukaryotes, which is consistent with the particle's fundamental role in packaging DNA into the nucleus (Turner, 1993, 2005). After prolonged digestion, the MNase degrades the chromatin to its fundamental repeating unit, called the nucleosome. In *M. incognita*, DNA fragment observed miccrococal nuclease digestion of 150 bp define the nucleosome that is consistent with what is known of the “primary structure” of chromatin with DNA fragment of length around the ~145–150 pb (Kornberg and Thomas, 1974; Luger et al., 1997; Rando, 2011). However, it is becoming clear that chromatin structures are not nearly as uniform and regular as previously assumed: nucleosomes vary in their histone protein components due to the incorporation of variant histones and PTMs (Luger et al., 2012; Turner, 2012). All PTMs, reversible for the most part, either change nucleosome structure directly by affecting histone proteins and DNA interactions or indirectly by recruiting binding proteins that act on the underlying chromatin structure, as has been proposed in the “histone code” hypothesis (Strahl and Allis, 2000). However, in spite of the high conservation of histone proteins along evolution, their degree of modification varies enormously according to the species, the developmental stage, the tissue and the cellular cycle phase (Strahl and Allis, 2000; Trojer and Reinberg, 2007; Luger et al., 2012; Turner, 2012). Here, we provided evidences for a “canonical nucleosome structure” in *M. incognita* with recognition of both unmodified and modified histone H3 as well as modified histone H4. Both euchromatin and heterochromatin regions contain specific PTMs and binding proteins. For instance, in *C. elegans*, heterochromatin is enriched in trimethylated H3 histones at lysine 56 (H3K56Me3) and lysine 9 (H3K9Me3; Jack et al., 2013). Ultimately, to identify features of transcriptional networks that regulate developmental processes, ChIP-sequencing was completed genome-wide in *C. elegans* (Niu et al., 2011). We expect to build on this foundation, together with this preliminary study, for further evaluation of transcriptional regulatory mechanisms during *M. incognita* development.

In addition, it is to be noted that nematodes could be divided in monocentric species and holocentric species (Subirana and Messeguer, 2013). *C. elegans* as well as *M. incognita* belong to the latter in which the centromeric function spreads out over the whole chromosomes (Triantaphyllou, 1983; Dernburg, 2001; Melters et al., 2012). Moreover, changes in chromosome number and structural rearrangements have been reported in Meloidogyne species (Triantaphyllou, 1981; Castagnone-Sereno, 2006; Abad et al., 2008). Being able to study position and histone protein components of *M. incognita* nucleosomes can be used to better understand diverse biological processes including DNA replication, recombination, mutation and repair. Because of the intimate relationship between nucleosome locations and these processes, we anticipate that ChIP-seq experiments in *M. incognita* will shed more light on genome evolution that seems to be crucial for an organism whose mode of reproduction is obligatory mitotic parthenogenesis (apomixis).

One major role for epigenetic variation in evolution would be to promote phenotypic variability, and allow populations to widely explore new environmental conditions. However, the detailed mechanisms thanks to which parasites evade host immunity for instance are poorly understood. *M. incognita* constitutes an ideal model to study these mechanisms, especially as inheritance of the nematode virulence does not segregate in accordance with Mendel's laws together with asexual multiplication in host plants (Castagnone-Sereno et al., 1994). Because clones can be obtained, genetic variability is reduced. As a result, *M. incognita* provides support for the study of epigenetic heredity distinct from the canonical Mendelian rules. Recently, it became apparent that parasitism success is partly accomplished by epigenetic means in several parasites, including, among others, protozoans Plasmodium and trematodes Schistosoma. More especially, PTMs plays a key role in the regulation of gene transcription of these parasites (Mourão et al., 2012; Perrin et al., 2013). For instance, it has been conclusively shown that PTMs, especially acetylations and methylations, and the propagation of heterochromatin away from the telomeres control the transcriptional switching between VAR genes that will trigger antigenic variation and enable immune evasion by the parasite, *Plasmodium falciparum* (Freitas-Junior et al., 2005; Chookajorn et al., 2007; Malmquist et al., 2012; Jiang et al., 2013). In *Schistosoma mansoni*, N-ChIP-seq experiments have been successfully performed to compare chromatin structure at different stages of the parasite life cycle, on different strains and on different sexes (Caby and Pierce, 2009; Cosseau et al., 2009; Lepesant et al., 2011, 2012). These advances in understanding *S. mansoni* epigenetic regulation have lead to the identification of promising new targets for the development of new drug to combat schistosomiasis (Pierce et al., 2012; Lancelot et al., 2013; Marek et al., 2013).

The importance of PTMs in regulating crucial developmental processes in parasites combined with the recent resolution of the genome sequences of *M. incognita* make the study of epigenetic machinery possible and susceptible to break new ground.

## Conclusion

For the first time in *M. incognita*, this study details DNA methylation and nucleosome structure, carriers of epigenetic information. Genomic DNA of *M. incognita* is not methylated. The general characteristics of nucleosome structure that we observed in the *M. incognita* genome are in accordance to what epigenetic studies have reported from other invertebrate species. Histone modifications, important markers of function and chromatin state, were identified in *M. incognita*. This study opens the way for analyzing epigenetic mechanisms in *M. incognita* at a whole-genome scale to identify new biological processes involved in the generation of phenotypic variation in absence of sexual reproduction in an ecological and economical important model.

## Author contributions

Conceived and designed the experiments: Céline Cosseau, Christoph Grunau, Laetitia Perfus-Barbeoch, Pierre Abad, Philippe Castagnone-Sereno. Performed the experiments: David Roquis, Laetitia Perfus-Barbeoch, Loris Pratx, Michael Reichelt, Sara Fneich. Analyzed the data: Céline Cosseau, Christoph Grunau, Laetitia Perfus-Barbeoch, Pierre Abad, Philippe Castagnone-Sereno. Wrote the paper: Céline Cosseau, Christoph Grunau, David Roquis, Laetitia Perfus-Barbeoch, Michael Reichelt, Pierre Abad, Philippe Castagnone-Sereno, Sara Fneich.

### Conflict of interest statement

The authors declare that the research was conducted in the absence of any commercial or financial relationships that could be construed as a potential conflict of interest.

## Supplementary material

**Supplementary material** : Links to public databases.

Expressed sequenced tags (EST) of *M. incognita* from NCBI-dbEST: http://www.ncbi.nlm.nih.gov/nucest/?term=meloidogyne+incognita

Primer3 web-interface: www.bioinformatics.nl/primer3plus/

*M. incognita* whole proteome: http://www6.inra.fr/meloidogyne_incognita/Genomic-resources/Blast

Supplementary Figure 1 **Quality and specificity controls for qPCR analysis**. **(A)** Electrophoresis gel of 150 bp genomic DNA amplified with primers specifically designed for ChIP validation. Markers 1 and 2 exhibit regularly spaced bands ranging from 200 to 10,000 bp and 100 to 1000 bp, respectively. **(B)** Primer efficiency was assessed on increasing genomic DNA concentration (from 0 to 1 ng/ul): increase in fluorescence intensity is proportional to the increase in amplicon concentration. **(C)** Melting curves and negative first derivative of the melting-curve: presence of a single homogeneous melt peak confirms specific amplification.

Supplementary Figure 2 **Western blots showing non-specific signals in *M. incognita* J2s**. **(A)** Anti-H3K4Me3 or Anti-H3K9Me3 antibodies. **(B)** Anti-H4PentaAc antibody. **(C)** Anti-H3K27Me3 or Anti-H3K36Me2 antibodies.

Supplementary Figure 3 **Electrophoresis gel of *M. incognita* eggs DNA after 0, 2, 4, 5, 6, and 7 min MNase (30U)**. Marker 1 and 2 exhibit regularly spaced bands ranging from 200 to 10,000 bp and 100 to 1000 bp, respectively.
