# Supplementary material for: Elucidating the molecular bases of epigenetic inheritance in non-model invertebrates: the case of the root-knot nematode Meloidogyne incognita
Source: Front Physiol. 2014 Jun 6;5:211. doi: 10.3389/fphys.2014.00211 (PMC4047830; doi:10.3389/fphys.2014.00211)
Supplement: Supplementary file 1 [file DataSheet1.DOCX]

**Supplementary Material**: ChIP-seq procedure for non-model organisms

This protocol is available on open-access: <http://194.167.139.26/cgrunau/methods/native_chip_sm.html>

ChIP-seq procedure – **Prepare the following solutions**

- 1 M KCl, autoclave
- 5 M NaCl, filter and autoclave
- 1 M MgCl, autoclave
- 1 M Tris/Cl pH 7.4 - 7.6, autoclave
- 0.5 M EDTA, autoclave
- 1 M CaCl_2_, 10 ml, autoclave
- 100 mM DTT, 1ml (store at -20°C)
- Roche Complete Protease Inhibitor (Reference: 11 697 498 001)
- 2.5 M Sodium butyrate (Reference: Sigma B5887 1g; store at 4°C)
- 25 mM PMSF in isopropanol, 10 ml (store at -20°C)
- 15 U/µl Micrococccal nuclease (MNase; Reference: USB 70196Y) in sterile 50% glycerol, aliquot to ~10µl (store at -20°C)
- Protein A - sepharose CL-4B (Reference: Sigma P3391 250mg; store at 4°C)
- agarose gel loading buffer
- 20% SDS
- 20 g/l glycogen solution (store at -20°C)
- 2% NaN_2_ in water (store at 4°C)
- micro-dialysis units (Reference: Slide-a-Lyzer 3500 D cut-off, Pierce 69550).

| Preparation of protein A - sepharose |
| --- |
| 1. weight 250 mg protein A - sepharose in 15 ml falcon tube 2. wash with 10 ml sterile water 3. centrifuge 10 min at 4000 rpm 4. remove supernatant 5. repeat step 3 - 4 four times 6. add sterile water to 5 ml 7. optional: add NaN_2_ to 0.02%   250 mg Protein A - sepharose swells to approx. 1 ml gel and binds approx. 20 mg human IgG. You will need 50 µl of the protein A sepharose homogeneously mixed in its 5 ml water volume per ChIP. |

| Optional if micro dialysis units are not available: preparation of dialysis tubing |
| --- |
| 1. cut tubing (e.g. VWR international dialysis tube 0.5 mm) into pieces of 10 - 20 cm length 2. boil for 10 min in a large volume of 2% (w/v) sodium bicarbonate and 1 mM EDTA 3. rinse the tubing thoroughly with distilled water 4. boil for 10 min in 1 mM EDTA 5. cool and store in this solution at 4°C 6. before use, wash tubing inside and outside with distilled water |

ChIP-seq procedure – **Day 1**

- reserve centrifuge and cool down to 4°C
- prepare a 2% 0.5x TBE agarose gel with 20 µl slots
- preheat a water bath to exactly 37°C
- prepare the following solutions with autoclaved distilled water

| 2X base buffer | | |
| --- | --- | --- |
| 6 ml | 1 M KCl | 60 mM final 1x |
| 0.3 ml | 5 M NaCl | 15 mM |
| 0.5 ml | 1 M MgCl2 | 5 mM |
| 20 µl | 500 mM EDTA | 0.1 mM |
| 1.5 ml | 1 M Tris/Cl | 15 mM |
| to 50 ml | water |  |
| 2 | Roche protease inhibitor tabletts | |

| Buffer1 (0.3 M sucrose) | |
| --- | --- |
| 2.58 g | sucrose |
| 12.5 ml | 2x base buffer |
| 50 µl | sodium butyrate |
| 100 µl | PMSF |
| 125 µl | DTT |
| to 25 ml | water |

| Buffer 2 | |
| --- | --- |
| 10 ml | buffer 1 (0.3 M sucrose) |
| put on 37°C to allow NP40 to be pipetted into the buffer | |
| 80 µl | NP40 (cut pipette tip) |
| put on 37°C to fully dissolve NP40 and put on ice | |

| Buffer 3 (1.2 M sucrose) for 3 cell samples | |
| --- | --- |
| 20.55 g | sucrose |
| 25 ml | 2x base buffer |
| 100 µl | sodium butyrate |
| 200 µl | PMSF |
| 250 µl | DTT |
| to 50 ml | water |

| MNase digestion buffer | |
| --- | --- |
| 1.1 g | sucrose |
| 0.5 ml | Tris/Cl |
| 80 µl | PMSF |
| 40 µl | MgCl2 |
| 20 µl | sodium butyrate |
| 10 µl | CaCl2 (essential for the enzyme) |
| to 10 ml | water |
| put at 37°C | |

| Dialysis buffer | |
| --- | --- |
| 1mM Tris/Cl, 200 µM EDTA, 200 µM PMSF, 5 mM sodium butyrate | |
| 50 µl | Tris/Cl |
| 20 µl | EDTA |
| 400 µl | PMSF |
| 100 µl | sodium butyrate |
| to 50 ml | water |

- put all buffer solutions on ice (except MNase buffer)
- cell lysis
  - aliquote 1500 sporocysts, miracidia or 10-20 adults (stored at -80°C or in liquid nitrogen)
  - adults:
    - remove excess liquid (if any) and resuspend in 1 ml buffer 1, add 1 ml buffer 2 (lysis buffer) and transfer to Dounce
    - homogenize for 3 min with Dounce (pestle A) on ice
    - put on ice 7 min
  - sporocysts or miracidia:
    - centrifuge into Eppendorf tubes, rinse storage tubes with PBS to recover all larvae
    - centrifuge at 4000 rpm, 10 min, 4°C
    - remove supernatant
    - resuspend completely in 1 ml buffer 1
    - do not add human lymphoblast cells as carrier
    - add 1 ml buffer 2 (lysis buffer) and homogenize for 3 min with Dounce (pestle A) on ice
    - put on ice 7 min
  - fill 8 ml buffer 3 into a 50 ml corex centrifugation tube
  - overlay the 8 ml buffer 3 with 1 ml cell suspension so that the tubes are ready for centrifugation 15 min (sporocysts) of 10 min (adults) after buffer 2 has been added to the cells
  - disturb a little bit the interface
  - use 2 corex tubes for the sporocysts sample that is in 2 ml buffer 1+2
  - mark tubes at the exterior side (to know where to look for the nuclei)
  - centrifuge 8500 rpm 20 min 4°C
  - carefully remove supernatant completely
- MNase digestion
  - resuspend pellet in 1 ml MNase digestion buffer
  - aliquot 500 µl of this suspension in 1.5 ml Eppendorf tubes
  - add 1 µl MNase (15 U) and incubate 4 min at 37°C
  - to stop the reaction add 20 µl 0.5 M EDTA to each 500 µl MNase digest and put the tube on ice
  - centrifuge 13000 g 10 min 4°C
  - transfer the supernatant to a new tube (S1) and keep the pellet (P1)
  - store S1 at -20°C
  - quantify chromatin in S1 by measuring OD at 260 nm in disposable cells against MNase buffer (In general we find about 50 µg/ml DNA in the undiluted S1, OD260/280 values can be bad because there is a lot of protein in the solution. DNA quantification is therefore not precise but sufficient for reproducibility.)
- Dialysis of P1
  - humidify Slide-a-Lyzer with 50µl dialysis buffer
  - resuspend the pellet P1 in 100 µl dialysis buffer and dialyze overnight at 4°C against 50 ml dialysis buffer with gentle stirring

ChIP-seq procedure – **Day 2**

- - the next day, transfer dialysed sample to Eppendorf tubes and...
    - centrifuge 13000 g 10 min 4°C
    - transfer the supernatant to a new tube and repeat the centrifugation 2 times
    - supernatant is fraction S2
  - yesterdays supernatant S1...
    - in parallel with the dialyses sample, centrifuge 13000 g 10 min 4°C
    - transfer the supernatant into a new tube and repeat this centrifugation 2 times
  - these triple centrifugations are IMPORTANT! They reduce the unspecific background!
  - use 50 µl of S1 and S2 for phenol/chlorofrom extraction, centrifuge and load 20 µl of supernatant on 2% 0.5x TBE gel (100V, 25 min)
- incubation with antibody
  - Ideally, the antibody should be in excess over the protein you want to precipitate. The antigen/antibody ration must be determined experimentally for each antibody . Prepare a dilution series of your chromatin in MNase buffer starting with 20 - 40 µg DNA for histon ChIP.
  - Add appropriate amounts of stock solutions to generate the

| antibody incubation buffer | |
| --- | --- |
| NaCl | 150 mM |
| Tris/Cl | 20 mM |
| sodium butyrate | 20 mM |
| EDTA | 5 mM |
| PMSF | 100 µM |

- - you can download an Excel worksheet for calculation [here (v0.1)](http://194.167.139.26/cgi/download_dump.cgi?file_name=compensation_buffer_150mM.xls) or [here (v1.0)](http://194.167.139.26/cgi/download_dump.cgi?file_name=Compensation_buffer_template_v1_0.xlsx)
  - dissolve chromatin from S1 (and S2 if you have dialyzed) in 1 ml buffer
  - add about 2 µg antibody
  - incubate overnight at 4°C on a rotating wheel

ChIP-seq procedure – **Day 3**

- precipitation
  - prepare 50 µl of protein A - sepharose for each tube
  - wash the beads to remove NaN2: short spin, remove supernatant and replace with equal volume of sterile water
  - add 50 µl of protein A - sepharose to each tube
  - incubate at least 4 h at 4°C on a rotating wheel
  - prepare washing buffers (10 ml / tube) and cool down to 4°C:

| washing buffers A B C | | | 50 ml | 100 ml | 200 ml | 300 ml |
| --- | --- | --- | --- | --- | --- | --- |
|  | Tris/Cl | 50 mM | 2.5 ml | 5 ml | 10 ml | 15 ml |
|  | EDTA | 10 mM | 1 ml | 2 ml | 4 ml | 6 ml |
|  | sodium butyrate | 5 mM | 100 µl | 200 µl | 400 µl | 600 µl |
| washing buffer A | NaCl | 75 mM | 750 µl | 1.5 ml | 3 ml | 4.5 ml |
| washing buffer B | NaCl | 125 mM | 1.25 ml | 2.5 ml | 5 ml | 7.5 ml |
| washing buffer C | NaCl | 175 mM | 1.75 ml | 3.5 ml | 7 ml | 10.5 ml |

- - centrifuge chromatin/antibody mixture 10 min 4°C 11600 g
  - keep the supernatant in a 2 ml tube. This is the unbound fraction UB.
  - resuspend the pellet in approx. 1 ml washing buffer A and transfer into a 15 ml Falcon tube containing 9 ml washing buffer A
  - mix for 10 min on a rotating wheel at 4°C (speed 6)
  - centrifuge 10 min 4000 rpm 4°C and pour off supernatant
  - add 10 ml washing buffer B, mix for 10 min on a rotating wheel at 4°C and centrifuge 10 min 4000 rpm 4°C
  - pour off supernatant
  - add 10 ml washing buffer C, mix for 10 min on a rotating wheel at 4°C and centrifuge 10 min 4000 rpm 4°C
  - pour off supernatant
  - centrifuge 10 min 4000 rpm 4°C
  - remove remaining supernatant completely
  - resuspend pellet in 500 µl elution buffer

| elution buffer | | 10 ml | 20 ml |
| --- | --- | --- | --- |
| SDS (20% stock) | 1 % | 500 µl | 1ml |
| Tris/Cl | 20 mM | 200 µl | 400 µl |
| NaCl | 50 mM | 100 µl | 200 µl |
| EDTA | 5 mM | 100 µl | 200 µl |
| sodium butyrate | 20 mM | 80 µl | 160µl |
| PMSF | 100 µM | 40 µl | 80 µl |
| water |  | to 10 ml | to 20 ml |

- - transfer suspension to a 1.5 ml Eppendorf tube
  - incubate 15 min at RT on a rotating wheel
  - centrifuge 10 min 11600 g 18°C
  - transfer supernatant to a 1.5 ml Eppendorf tube
  - This is the bound fraction B.
- DNA extraction
  - extract DNA with phenol/chloroform from fractions B and UB
  - add 1 µl of a 20 g/l glycogen stock solution
  - add NaCl to 250 mM (26 µl and 52 µl) and add 1 volume isopropanol
  - put overnight at -20°C
  - precipitate by centrifugation and wash with 70% ethanol
  - dry the pellet and resuspend in 20 µl 10 mM Tris/Cl or qPCR grade water
  - use 1 µl of this DNA for PCR in 25 µl reactions (quantitative real-time PCR) or 10 µl (PCR)
