# Supplementary figures and images for: Elucidating the molecular bases of epigenetic inheritance in non-model invertebrates: the case of the root-knot nematode Meloidogyne incognita
Source: Front Physiol. 2014 Jun 6;5:211. doi: 10.3389/fphys.2014.00211 (PMC4047830; doi:10.3389/fphys.2014.00211)

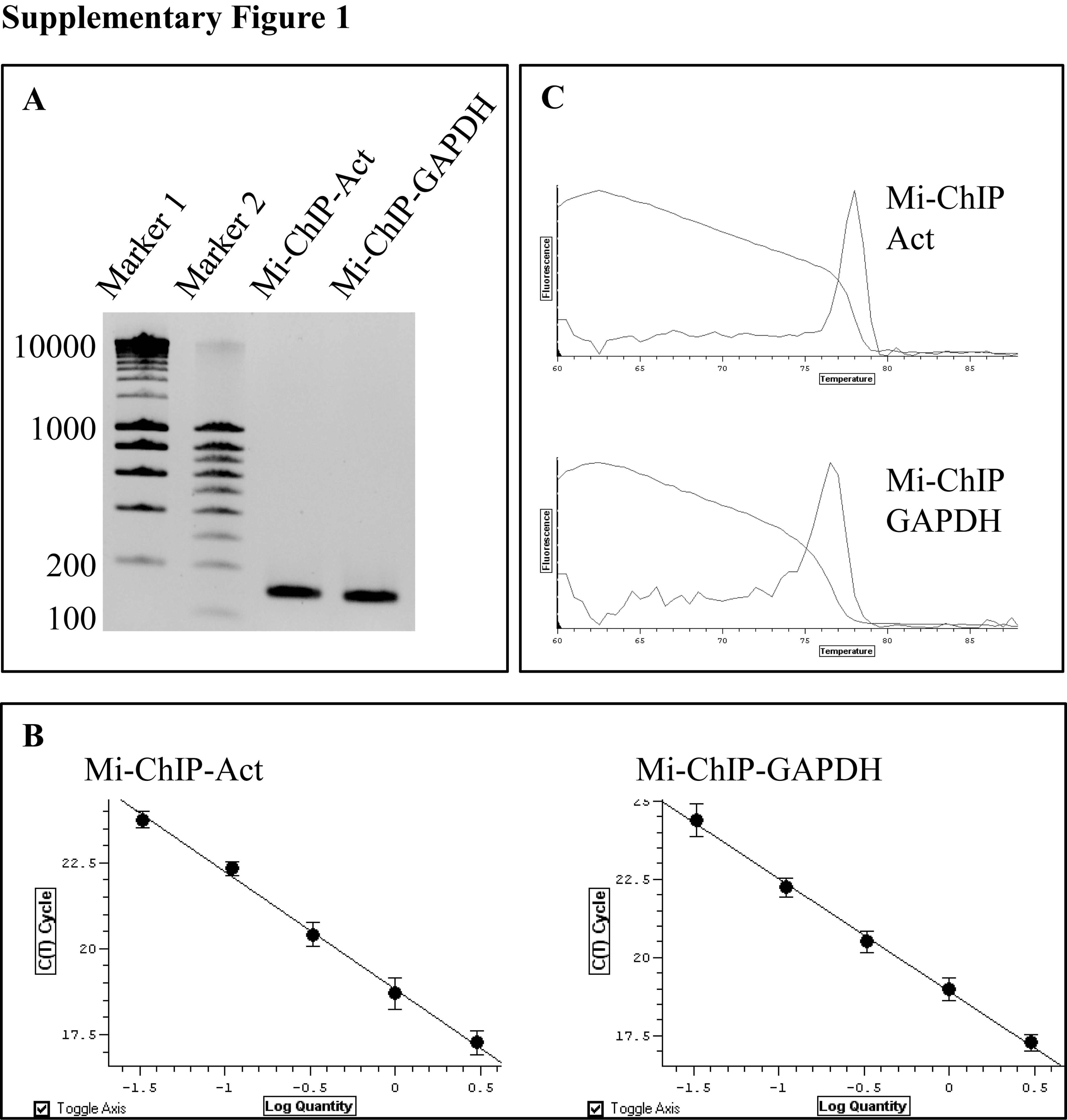

Supplement: Supplementary Figure 1 — Quality and specificity controls for qPCR analysis. (A) Electrophoresis gel of 150 bp genomic DNA amplified with primers specifically designed for ChIP validation. Markers 1 and 2 exhibit regularly spaced bands ranging from 200 to 10,000 bp and 100 to 1000 bp, respectively. (B) Primer efficiency was assessed on increasing genomic DNA concentration (from 0 to 1 ng/ul): increase in fluorescence intensity is proportional to the increase in amplicon concentration. (C) Melting curves and negative first derivative of the melting-curve: presence of a single homogeneous melt peak confirms specific amplification. [file Presentation_1.ZIP › Supplementary material/78141_Perfus-Barbeoch_Supp Fig 1.TIF]

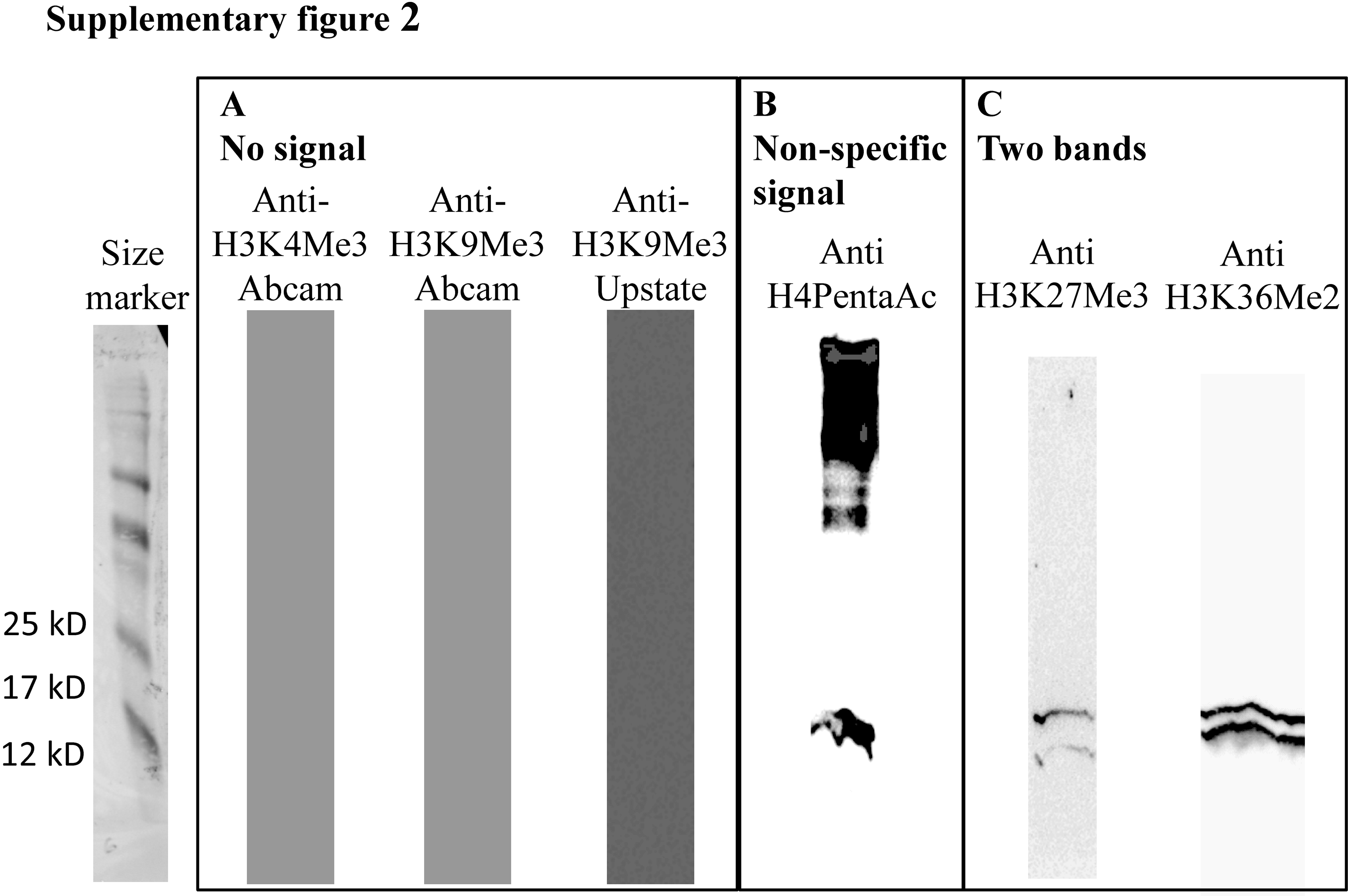

Supplement: Supplementary Figure 1 — Quality and specificity controls for qPCR analysis. (A) Electrophoresis gel of 150 bp genomic DNA amplified with primers specifically designed for ChIP validation. Markers 1 and 2 exhibit regularly spaced bands ranging from 200 to 10,000 bp and 100 to 1000 bp, respectively. (B) Primer efficiency was assessed on increasing genomic DNA concentration (from 0 to 1 ng/ul): increase in fluorescence intensity is proportional to the increase in amplicon concentration. (C) Melting curves and negative first derivative of the melting-curve: presence of a single homogeneous melt peak confirms specific amplification. [file Presentation_1.ZIP › Supplementary material/78141_Perfus-Barbeoch_Supp Fig 2.TIF]

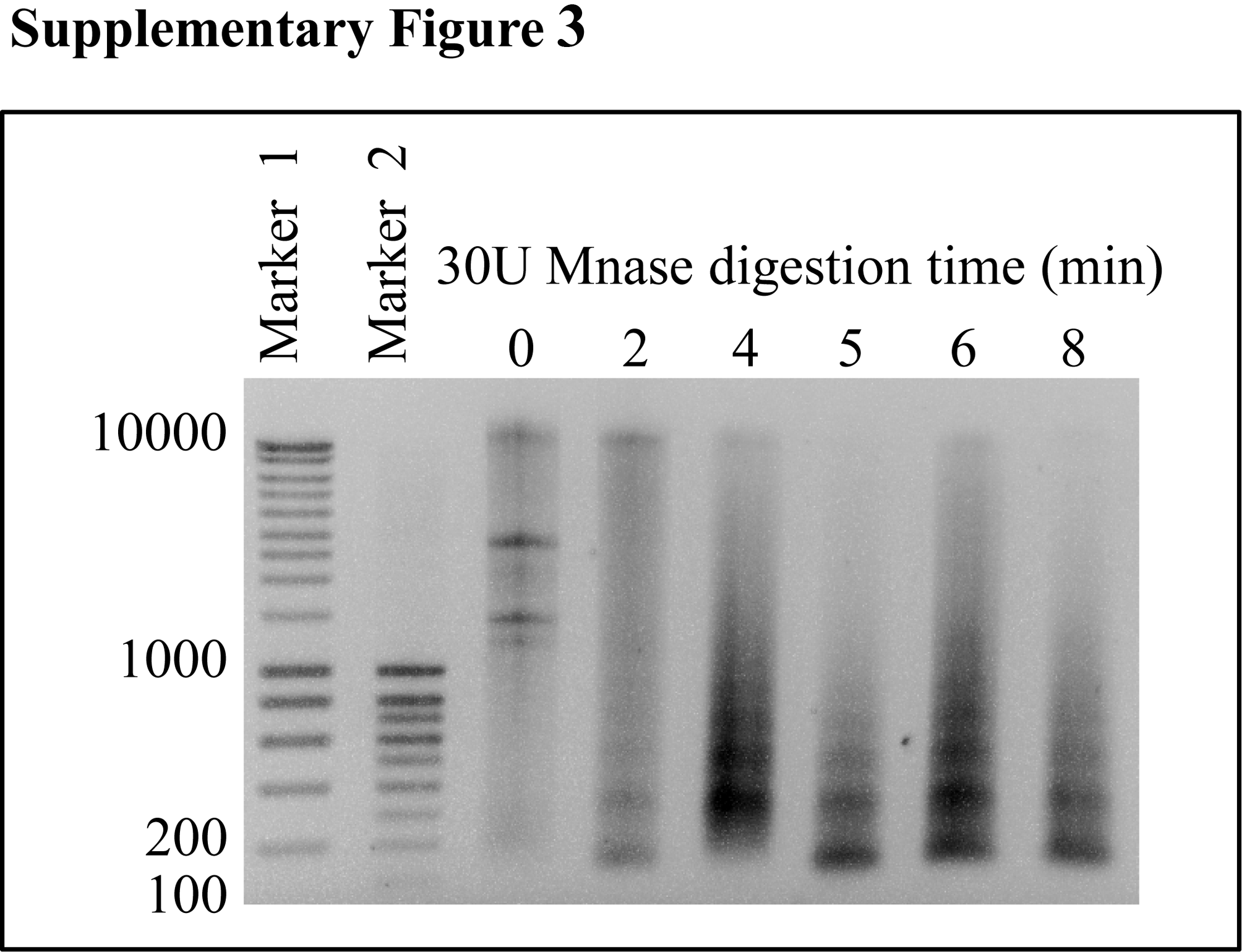

Supplement: Supplementary Figure 1 — Quality and specificity controls for qPCR analysis. (A) Electrophoresis gel of 150 bp genomic DNA amplified with primers specifically designed for ChIP validation. Markers 1 and 2 exhibit regularly spaced bands ranging from 200 to 10,000 bp and 100 to 1000 bp, respectively. (B) Primer efficiency was assessed on increasing genomic DNA concentration (from 0 to 1 ng/ul): increase in fluorescence intensity is proportional to the increase in amplicon concentration. (C) Melting curves and negative first derivative of the melting-curve: presence of a single homogeneous melt peak confirms specific amplification. [file Presentation_1.ZIP › Supplementary material/78141_Perfus-Barbeoch_Supp Fig 3.TIF]
